# Identification of both copy number variation-type and constant-type core elements in a large segmental duplication region of the mouse genome

**DOI:** 10.1186/1471-2164-14-455

**Published:** 2013-07-08

**Authors:** Juzoh Umemori, Akihiro Mori, Kenji Ichiyanagi, Takeaki Uno, Tsuyoshi Koide

**Affiliations:** 1 Mouse Genomics Resource Laboratory, National Institute of Genetics, 1111 Yata, Mishima, Shizuoka 411-8540, Japan; 2 Transdisciplinary Research Integration Center, Research Organization of Information and Systems, 4-3-13 Toranomon, Minato-ku, Tokyo 105-0001, Japan; 3 Program in Gene Function and Expression, University of Massachusetts Medical School, Worcester, MA 01605, USA; 4 Division of Epigenomics and Development, Department of Molecular Genetics, Medical Institute of Bioregulation, Kyushu University, Fukuoka 812-8582, Japan; 5 National Institute of Informatics, Hitotsubashi 2-1–2, Chiyoda-ku, Tokyo 101-8430, Japan; 6 Department of Genetics, SOKENDAI, Mishima, Shizuoka 411-8540, Japan; 7 Present address: Division of Systems Medical Science, Institute for Comprehensive Medical Science, Fujita Health University, Toyoake, Aichi 470-1192, Japan

**Keywords:** Comparative genome hybridization array, Repetitive element, Retrotransposon, Mouse genome, Homology search

## Abstract

**Background:**

Copy number variation (CNV), an important source of diversity in genomic structure, is frequently found in clusters called CNV regions (CNVRs). CNVRs are strongly associated with segmental duplications (SDs), but the composition of these complex repetitive structures remains unclear.

**Results:**

We conducted self-comparative-plot analysis of all mouse chromosomes using the high-speed and large-scale-homology search algorithm SHEAP. For eight chromosomes, we identified various types of large SD as tartan-checked patterns within the self-comparative plots. A complex arrangement of diagonal split lines in the self-comparative-plots indicated the presence of large homologous repetitive sequences. We focused on one SD on chromosome 13 (SD13M), and developed SHEPHERD, a stepwise *ab initio* method, to extract longer repetitive elements and to characterize repetitive structures in this region. Analysis using SHEPHERD showed the existence of 60 core elements, which were expected to be the basic units that form SDs within the repetitive structure of SD13M. The demonstration that sequences homologous to the core elements (>70% homology) covered approximately 90% of the SD13M region indicated that our method can characterize the repetitive structure of SD13M effectively. Core elements were composed largely of fragmented repeats of a previously identified type, such as long interspersed nuclear elements (LINEs), together with partial genic regions. Comparative genome hybridization array analysis showed that whereas 42 core elements were components of CNVR that varied among mouse strains, 8 did not vary among strains (constant type), and the status of the others could not be determined. The CNV-type core elements contained significantly larger proportions of long terminal repeat (LTR) types of retrotransposon than the constant-type core elements, which had no CNV. The higher divergence rates observed in the CNV-type core elements than in the constant type indicate that the CNV-type core elements have a longer evolutionary history than constant-type core elements in SD13M.

**Conclusions:**

Our methodology for the identification of repetitive core sequences simplifies characterization of the structures of large SDs and detailed analysis of CNV. The results of detailed structural and quantitative analyses in this study might help to elucidate the biological role of one of the SDs on chromosome 13.

## Background

Copy number variation (CNV) of genomic segments is a common phenomenon that affects approximately 12% and 10.7% of the human and mouse genomes, respectively [1-5]. Comprehensive genomic analyses have shown that CNV sequences often overlap and form clusters of variable regions [3,6,7]. These regions, known as CNV regions (CNVRs), are associated with variations in gene expression and phenotype [3,5,7-15]. Frequently, CNVRs of intermediate size and larger (>10 kbp) are associated with segmental duplications (SDs) in the human and mouse genomes [3,6,7,16]. An SD is defined as a block of highly homologous (>90%) duplicated genomic DNA that, in the human genome, can range from 1 kbp to several hundred thousand bp [13,15]. In the mouse genome, SDs can be as large as 1 Mbp in size [17,18]. Many of the large SDs contain repetitive sequences with ambiguous borders and copy numbers that vary among strains. These sequences are called complex CNVRs [7]. A previous study proposed that CNVRs are associated with differences in gene expressions among strains, possibly through changes of local chromatin structures in CNVRs [7]. Previous studies identified SD regions through systematic analysis of the mouse genome and characterized CNV in these regions [17,18]. However, the detailed character of the repeating unit and the structure of the duplication pattern remained to be resolved. To better understand the evolution of SDs and the biological role of CNVRs, the repetitive structure of SDs must be elucidated in more detail. In this study, we aimed to identify repetitive “core elements” as well as copy numbers of the elements and the detailed structure of large SDs in the mouse genome. Core elements were defined as consensus sequences of repetitive sequences and were expected to be the basic units that formed SDs.

We characterized the organization and variation in copy number of core elements in one of the large SDs on chromosome 13 in mice. The strategy implemented in this study involved four steps: (i) self-comparison of the DNA sequences of entire mouse chromosomes (self-comparative-plot analysis) using the high-speed and large-scale-homology search algorithm, Similarity/Homology Efficient Analyze Procedure (SHEAP), to identify candidate SDs [19], (ii) identification of core elements and description of the repetitive structure of the SD, using the newly developed stepwise *ab initio* method, blast-based Systematic analysis of HErPlot to Extract Regional Distinction (SHEPHERD), (iii) comparison of the CNV found in the core elements among mouse strains by comparative genome hybridization array (aCGH), and (iv) characterization of core elements that contain CNV (CNV type) and those that do not (constant type).

## Results

### Detection of segmental duplications by SHEAP

**Figure 1:**
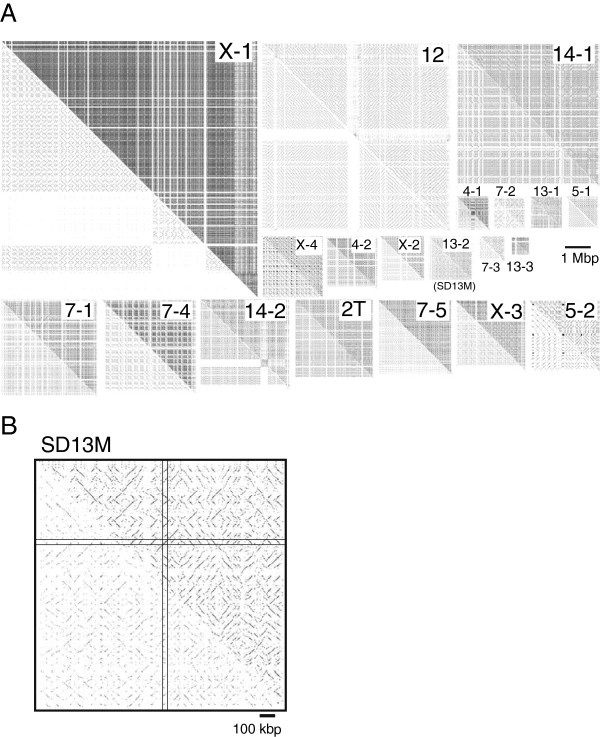
**Tartan-checked structure of SDs visualized using SHEAP. **Diagonal lines aligned in the same column or row represent repetitive sequences. The lower left triangle of each panel shows a self-plot of the sequence after known repeat sequences have been masked using RepeatMasker. Each of the upper right triangles shows a self-plot of the intact sequence. **(A) **All of the SDs detected by SHEAP for all chromosomes except Chr Y. Rough estimates of the genomic positions of segmental duplications were: Chr. X-1, 33,454–43,954 (10,500); Chr. 12, 25,264–32,863 (7,599); Chr. 14–1, 8,647–14,424 (5,776); Chr. 7–1, 10,860–14,710 (3850); Chr. 7–4, 24,250–28,000 (3,750); Chr. 14–2, 45,553–49,106 (3,553); Chr. 2 T, 177,898–181,042 (3,144); Chr. 7–5, 34,938–37,919 (2,981); Chr. X-3, 122,800–125,582 (2,782); Chr. 5–2, 96,512–99,314 (2,802); Chr. X-4, 146,542–148,959 (2417); Chr. X-2, 53,082–54,812 (1730); Chr. 4–2, 61,950–64,050 (2,100); Chr. 13–2, 67,076–68,893 (1817); Chr. 4–1, 42,923–44,207 (1284); Chr. 7–2, 11,339–12,249 (910); Chr. 13–1, 62,919–64,168 (1,249); Chr. 5–1, 11,973–13,221 (1,249); Chr. 7–3, 12,327–13,309 (982); Chr. 13–3, 68,702–69,433 (730). Numbers in parentheses indicate the sizes of SDs (kbp). **(B)** Higher magnification for the self-plot of SD13M, located from 65,370,000 to 67,000,000 on Chr 13. The diagonal lines from top left to bottom right, which indicate a complete match between SD13M sequences, were eliminated by the algorithm. To remove redundant and overlapping sequences from 2,638 repetitive sequences, the sequences represented by diagonal lines that were located in the same column or in the same row (enclosed by two lines in the horizontal and vertical directions, respectively) were eliminated.

In order to detect candidate SDs, we conducted self-comparative-plot analysis of mouse genome sequences using the SHEAP method. For certain chromosomes, the output of the self-comparative-plot analysis contained square dark patches (Additional file 1, arrowheads). Additional file 2 shows an example of the output from the self-comparative-plot for an entire chromosome (chromosome 13) with a dark background. Further magnification of these patches revealed a tartan-checked pattern with a complex arrangement of diagonal split lines, which indicated the presence of homologous repetitive sequences (Figure 1A). We selected candidates for large SDs as the regions that visually showed tartan-checked patterns larger than 500 kbp. These large SDs comprised repetitive sequences in both forward and reverse orientations, and were arranged in various patterns. All mouse chromosomes were analyzed except chromosome Y. Of the 20 chromosomes analyzed, eight contained large SD regions. After previously known repetitive elements had been masked using RepeatMasker [20], the number of diagonal lines obtained in the self-comparative plot described above was reduced in a large proportion of the SDs (Figure 1A, lower left). This result indicated that most of the SDs contained a large number of known repetitive elements (Table 1), as reported previously [7,18]. We focused on one of the SDs, named SD13M, which is spans nucleotide 67,076,000 to 68,893,000 on chromosome 13 (Figure 1 and Additional file 1 (red arrowhead)). SD13M was chosen because this SD contains a wide range of repeats in both forward and reverse orientations. Furthermore, a previous study reported difficulty in transferring the chromosomal segment including SD13M from one mouse strain, MSM/Ms (MSM), to another, C57BL/6J (B6), in the course of establishing a consomic strain [21]. Therefore, SD13M may have an important biological role. The reduction in the number of diagonal lines in this region after the masking of known repeats (Figure 1B) indicates that this region also contained a large number of known repetitive elements. The proportion of known repetitive elements in SD13M was similar to those of other SDs (Table 1).

**Table 1:** Comparison of the proportion of known repeats in SD13M with the values for the entire genome and average values for SDs

**Repeat**	**Ratio in SD13M (%)**	**Segmental duplication average (%) **[18]	**Whole genome average (%) **[18]
DNA	0.18	0.36	0.86
LINE	23.9	34.4	20.3
Low_complexity	0.82	0.58	0.79
LTR	18.3	19.5	10.2
Satellite (MMSAT4)	1.92	0.32	0.05
Simple_repeat	1.94	1.66	2.47
SINE	5.20	3.80	7.42
snRNA	0.00	0.01	0.01
tRNA	0.01	0.01	0.01
Unknown (MurSatRep)	5.97	0.54	0.05
Total	58.2	61.2	42.2

### Identification of core elements for SD13M

**Figure 2:**
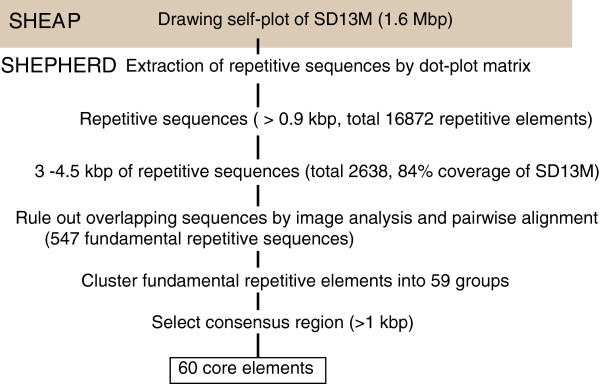
**Flowchart of core element identification for analysis of CNVs. **The figure provides an overview of the steps used to identify core elements.

We defined “fundamental repetitive sequences” as sequences that covered most of the repetitive structure of SD13M and could be used to extract core elements. For the identification of core elements, we developed SHEPHERD (Figure 2), a stepwise *ab initio* method that is designed to extract longer repetitive elements than previous methods [22-27], and involves the following three steps:

#### ***Extraction of fundamental repetitive sequences from a self-comparative-plot matrix***

**Figure 3:**
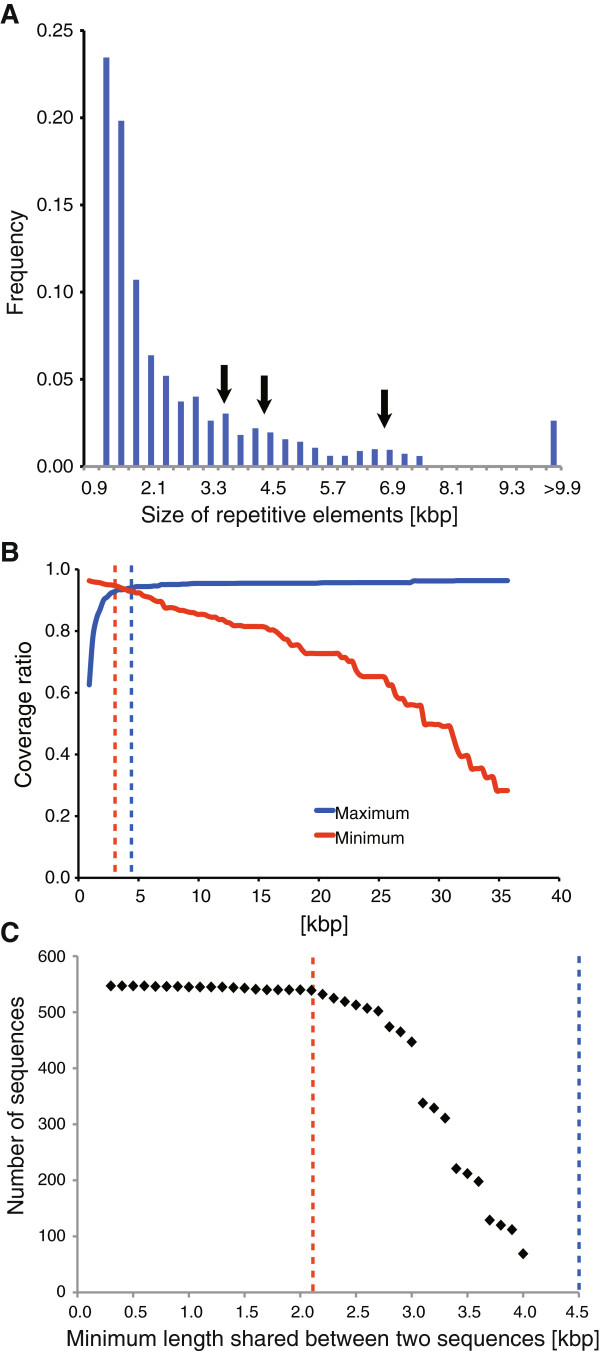
**Determination of the sizes of fundamental repetitive sequences for the identification of core elements. (A) **The distribution of 16,872 repetitive sequences. Four small peaks at 3, 3.6, 4.1, and 6–8.1 kbp are indicated by arrows. **(B) **The minimal lengths of the extracted repetitive sequences are plotted against the coverage ratio as a red line. The maximum lengths of the repetitive sequences are plotted against the coverage ratio as a blue line. The red line shows that SD13M is covered almost completely (>96%) by the extracted repetitive sequences, and the broken red line shows that 94% of SD13M is covered by repetitive sequences larger than 3 kbp. The broken blue line shows that most of SD13M (>94%) is covered by repetitive sequences smaller than 4.5 kbp. **(C) **The minimum lengths of the regions shared when two repetitive sequences were aligned are plotted against the number of sequences in which such regions are shared. The broken red line shows that 92% of pairs share 2.7 kbp of consensus sequences, whereas the broken blue line shows that 14% of pairs share 4.5 kbp of consensus sequences.

All diagonal lines in Figure 1B that comprised at least three consecutive dots [each dot represents 300 bp] were extracted from a self-comparative-plot of SD13M that consisted of a dot-plot matrix (Figure 1B). Consequently, 16,872 repetitive sequences, which ranged from 0.9 kbp to 79.5 kbp in length, were extracted (Figure 3A). Given that many repetitive sequences had different lengths (Figure 1B), we determined the most appropriate length for fundamental repetitive sequences to be one that was not too short but still covered most of the SD13M region. In terms of the distribution of the lengths of the repetitive sequences, one major peak was detected at approximately 1.2 kb, and four small peaks were identified at approximately 3, 3.6, 4.1, and 6–8.1 kbp, respectively (Figure 3A). When the minimum length was set to 0.9 kbp, the selected sequences covered more than 96% of the entire SD13M region (Figure 3B, red line). An increase of the minimum length resulted in a decrease in the coverage rate. When repetitive sequences of ≥3.0 kbp in length were selected, the coverage rate was still 94% of the entire SD13M (Figure 3B, red dotted line). In contrast, when the maximum length was decreased, the coverage rate decreased. When repetitive sequences of lengths ≤4.5 kbp were selected, the coverage of SD13M was greater than 94% (Figure 3B, blue dotted line). These analyses indicate that the most appropriate length of the fundamental repetitive sequences is between 3 kbp and 4.5 kbp.

Among the 16,872 repetitive sequences identified in SD13M, there were 2,638 sequences within the range of 3–4.5 kbp. To eliminate redundant and overlapping sequences from among these 2,638 repetitive sequences, we selected one sequence out of the 2,638 repetitive sequences and removed the other sequences represented by diagonal lines that were located in the same column or in the same row as that sequence in the self-comparative-plot map (Figure 1B). This step has the advantage of reducing machine loading given that the pairwise analysis that is most commonly used requires approximately 7 million (2638 * 2637) pairs, and thus require many days to complete. In contrast, by eliminating redundant and overlapping sequences from 2,637 sequences, the use of a self-comparative-plot map enables the analysis to be completed within a short period of time. After repeating this process for different sequences until all redundant sequences had been removed, 547 nonredundant and nonoverlapping repetitive sequences remained, which covered approximately 80% of the SD13M region. We defined these sequences as fundamental repetitive sequences.

#### ***Clustering of fundamental repetitive sequences***

We clustered the fundamental repetitive sequences into groups, such that there was the maximum redundancy in sequence similarity within each group, but the least possible redundancy between the groups. The overlap of these fundamental repetitive sequences was tested by pairwise alignments using bl2seq (without filtering option; –FF) [28,29]. We counted the number of sequences that overlapped with other sequences for different lengths of overlap (Figure 3C). The sequences of 92% of the total number of fundamental repetitive sequences shared at least 2.7 kbp in length, and 14% of the sequences shared 4 kbp with at least one other sequence. Given that most of the sequences that were shared had a size equivalent to that of fundamental repetitive sequences, which are between 3.0 and 4.5 kbp in length, the fundamental repetitive sequences could be classified into a smaller number of groups as follows. We considered that the most representative sequence in each group should have the highest number of matching counts with other sequences in the group, and that sequences similar to the representative sequence should belong to that group. As a result of this process, we clustered the 547 fundamental repetitive sequences into 59 groups (representative sequences).

#### ***Identification of core elements***

**Figure 4:**
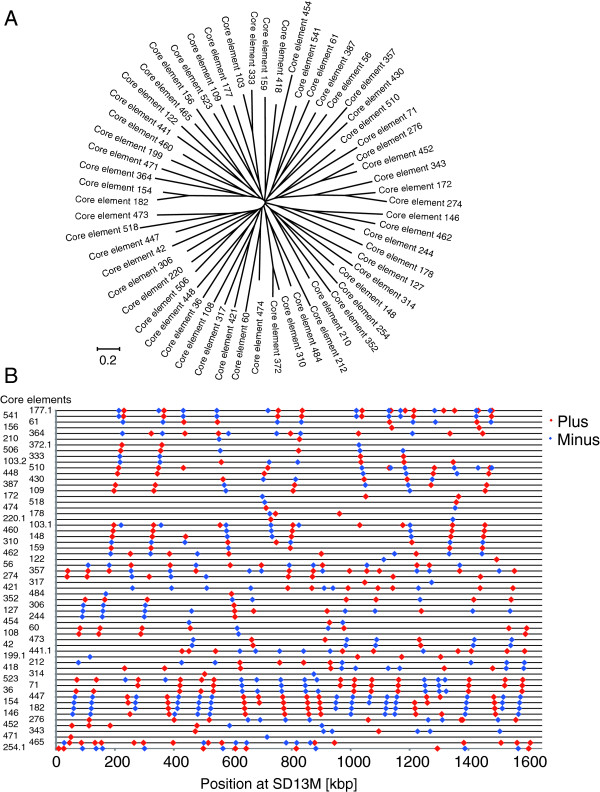
**Identification and characterization of core elements for SD13M. (A) ** MUSCLE analysis of identified core elements. The radial pattern with branches of similar length indicates that the levels of difference between the 60 types of core elements are similar. **(B) **Map of sequences homologous to each core element on SD13M. The locations and directions of homologous sequences for each core element are mapped on the horizontal line. Red and blue diamonds indicate positive and negative orientations, respectively.

In each clustered group, we identified core elements, which are defined on the basis of two criteria: (i) the length should be >1 kbp (without gaps) which is an arbitrary threshold, and (ii) the majority of sequences in each clustered group should share the consensus sequence of a given core element. Because one of the 59 groups contained two core elements, a total of 60 sequences was identified. The sequences of the core elements are shown in Additional file 3. Alignment of these core elements using MUSCLE revealed a radial pattern, which suggested that most of the core elements have similar divergence and that there is no strong homology among them (Figure 4A). Given that sequences homologous to the core elements (>70% homology) covered approximately 90% of the SD13M region, our method can characterize the repetitive structure of SD13M efficiently. The positions and directions of each core element within SD13M are shown in Figure 4B, and additional information on the core elements mapped in SD13M is summarized in Additional file 3. The pattern of distribution of the core elements also indicates the existence of a higher order of repeating units of various sizes because there are many places where multiple core elements are clearly located adjacent to each other (Figure 4B). However, because the borders between these regions are not clear, and the sizes vary from short to long, we could not characterize the larger repeating units further.

### Characterization of core elements

To characterize the core elements, we annotated them with RepeatMasker and with BLASTN using the RefSeqGene database. The known repeats and RefSeq sequences that were detected in each core element are listed in Additional file 4. As expected from the results of the self-plot analysis with masked sequences, all of the core elements contained at least a partial sequence of a known repeat, such as a long interspersed nuclear element (LINE), short interspersed nuclear element (SINE), or long terminal repeat (LTR) type of retrotransposon, as well as uncharacterized repeats such as MurSatRep1. The average proportion of known repeats in the core elements was 59.5% (Additional file 5), which indicated that core elements consisted largely of known repeats. Most of these known repeats were fragmented and overlapped with each other. One-third of the core elements (20 out of 60) contained partial sequences of RefSeq genes (Additional file 4). These partial sequences could be divided roughly into three types of reported or predicted genes: members of the zinc finger protein (*Zfp*) family, members of the vomeronasal 2 receptor (*Vmn2r*) family, and chromobox homolog 3 (*Cbx3*). The average proportion of the total lengths of these annotated gene-like sequences that was found in the core elements were 8% for *Zfp*, 16% for *Vmn2r*, and 37% for *Cbx3* (Additional file 6).

### CNV of core elements among mouse strains

**Figure 5:**
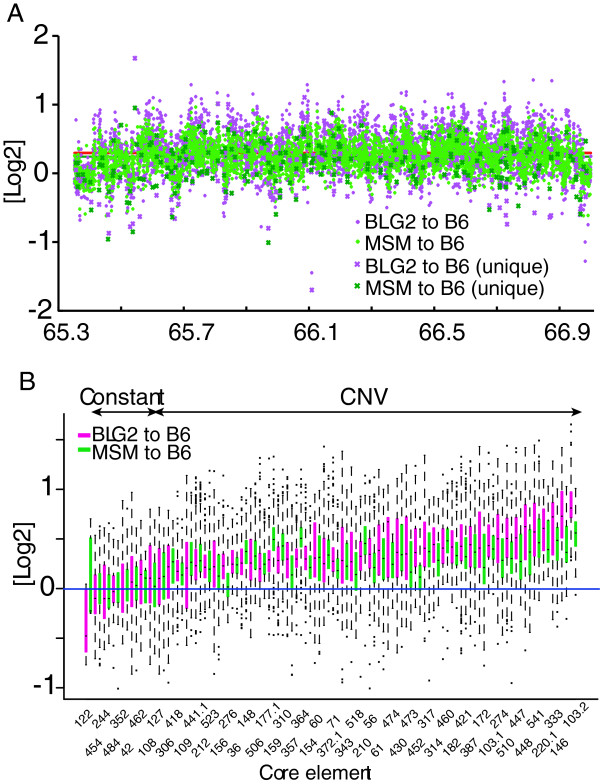
**Copy number analyses of core elements. (A) **Results of aCGH for the entire SD13M region. Mapped aCGH log2 values in SD13M that compare MSM (musculus subspecies group) or BLG2 (musculus) with B6 (domesticus) are shown by green and magenta diamonds, respectively. Relative copy numbers estimated by with unique probes are shown by X in the same colors. Average aCGH values for MSM compared with B6, and for BLG2 compared with B6 in the relevant regions are shown by red and green lines, respectively. **(B)** Boxplots of aCGH log2 values mapped on each core element. Boxplot of aCGH values for BLG2 compared with B6, and of MSM compared with B6 are shown in magenta and green, respectively. Dots indicate outliers. The boxplots are aligned in the order of the average aCGH values from the comparison of BLG and B6.

**Figure 6:**
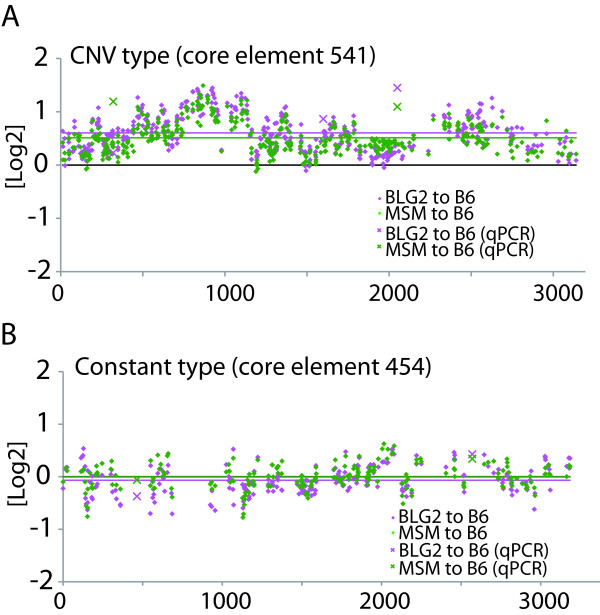
**Copy number analyses in representative core elements. **Mapped aCGH log2 values in the core elements that compare MSM (musculus subspecies group) or BLG2 (musculus) with B6 (domesticus) are shown by green and magenta diamonds, respectively. Relative copy numbers estimated by qPCR with unique probes using genomic DNA from BLG2, MSM, and B6 are shown by X in the same colors. Average aCGH values for MSM compared with B6, and for BLG2 compared with B6 in the relevant regions are shown by red and green lines, respectively. **(A) **Mapping of aCGH log2 values to a representative CNV-type core element (core element 541). Three relative values obtained by qPCR plotted around 1.0 (indicated by X), which shows that the copy number of core element 541 is higher in BLG2 and MSM than in B6. Primers for qPCR were designed for three regions of core element 541 (320–456, 1599–1693, and 2050–2162) (Additional file 5: Table S5). **(B) **Mapped aCGH values on a representative constant-type core element (core element 454) were distributed around zero. Furthermore, all the qPCR values plotted around zero (indicated by X). These results showed no CNV between B6 and MSM within core element 454.

The SD13M region comprises variously sized forward and reverse repetitive sequences, and was defined previously as a complex CNVR (Cahan et al. 2009). If core elements are sources of CNVRs as well as of SDs, they should correspond to distinctive strain-specific CNV. To test this hypothesis, we conducted aCGH analysis using a tiling array designed for the SD13M region to compare the copy numbers of the core elements between the mouse strains B6 and MSM, and between B6 and BLG2 (Figure 5A). The average copy numbers of the probes for the entire region of SD13M were greater in BLG2 and MSM than in B6 (Figure 5A, red and green horizontal lines, respectively). The aCGH values of a total of 9,929 probes were mapped on 53 out of the 60 core elements (see Methods). The mapped aCGH log2 values for BLG2 or MSM to the reference (B6) on each core elements are shown in Figure 5 and Additional file 5. In most core elements, the mapped aCGH values deviated from zero, and they had a similar distribution pattern throughout each core element. This observation indicated that the copy numbers of the core elements varied among strains. A t-test showed that 42 out of 53 core elements differed significantly in copy number, both between B6 and BLG2 and between B6 and MSM, and that three core elements differed significantly in only one pair of strains (Figure 5B, Additional file 5). These results indicated that most of the core elements displayed distinctive CNV among strains (CNV-type core elements), which suggests that they are the basic units related to the formation of both SDs and CNVs. The remaining eight core elements, which did not differ significantly in copy number between B6 and BLG2 or between B6 and MSM, should not be considered as CNV-type. These core elements were defined as being of constant type. The estimated copy numbers and the average aCGH values of the core elements are listed in Additional file 5. The mapping of a representative CNV-type (core element 541) and a constant-type (core element 454) is shown in Figure 6A and 6B, respectively.

The results of the aCGH were confirmed by quantitative PCR analysis using genomic DNA as the template with several sets of primers (Figure 6A and 6B, Additional files 7 and 8; see Methods). The qPCR analyses showed that the relative amount of core element 541 increased additively as the dosage of the MSM allele in a given consomic strain increased (Additional file 8A). Conversely, the relative amount of core element 454 remained almost constant when the dosage of the MSM allele increased in the same consomic strain (Additional file 8B). These results indicate that the copy number of core element 541 is greater in MSM than in B6, whereas the copy number of core element 454 does not differ between B6 and MSM.

### Comparison of constant-type and CNV-type core elements

**Figure 7:**
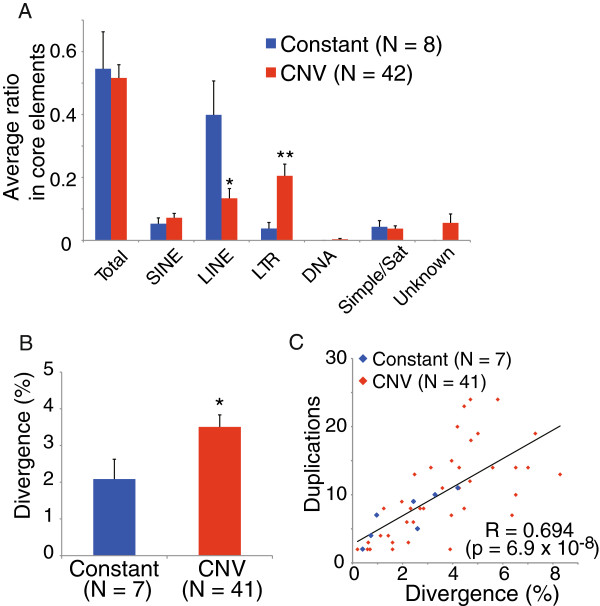
**Characteristics of core elements. (A) **Comparison of known repeats in CNV-type and constant-type core elements. Average proportions of the known repeats, classified into six categories (SINE, LINE, LTR, DNA transposon (DNA), simple/satellite repeats (Simple/Sat), and unknown repeats) in each core element were compared between the CNV and constant types of core element. *, P < 0.05; **, P < 0.001. Average proportions were calculated from the proportion of each type of repeat in each type of core element. **(B)** Comparison of divergence between CNV-type and constant-type core elements. Divergence is represented by percentage values for the number of mutations and insertions or deletions that were detected after pairwise comparison of’ sequences. **(C)** Correlation between the divergence of the sequences and the number of duplications of core elements. Core elements 177 and 254 were excluded from these analyses because their sequences are contained within core elements 541 and 244, respectively.

Next, we compared the sequence characteristics of the CNV and constant types of core element. The results of the annotation for both types of core element are listed in Table 2. All of the constant-type core elements are listed, together with the 10 CNV-type core elements that showed the greatest variation in copy number among strains. The CNV-type core elements preferentially contained various classes of LTR transposable elements, such as ORR1. Figure 7A shows the average proportion of known repeats, classified into six categories (SINE, LINE, LTR, DNA transposon, simple/satellite repeats, and uncharacterized repeats), in the core elements. Notably, the average proportion of LTR sequences was significantly higher in CNV-type core elements than in the constant type. In contrast, the average proportion of LINE sequences was significantly lower in CNV-type core elements than in the constant type. We investigated the divergence of homologous sequences in each group of core elements (Additional file 5). The average divergence of CNV-type core elements was greater than that of the constant type (Figure 7B). Furthermore, divergence was correlated with the number of duplications of the core elements in each group (Figure 7C). These results suggest that constant-type core elements emerged more recently than CNV-type core elements in SD13M.

**Table 2:** Annotation of known repeats in core elements with large CNV and without CNV

					**Expected copy number**^**c**^				
**Core element**	**Type**	**Length (bp)**	**Known repeats and RefSeq genes **^**a**^	**Ratio**^**b**^	**B6**	**BLG2**	**MSM**	**Probe (N)**	**Total length in**
									**B6 (bp)**
Core element 042	Constant	3000	LINE, *Vmn2r* , LTR	0.610	9	10	9	252	27000
Core element 108	Constant	3000	*Cbx3* , LTR, SINE	0.250	3	3	3	154	9000
Core element 127	Constant	2700	*Vmn2r* , LINE	0.158	12	13	13	196	32400
Core element 244	Constant	3247	LINE, *Vmn2r* , SINE	0.563	8	8	8	68	25976
Core element 352	Constant	3900	LINE,LTR, Simple, SINE	0.911	8	8	8	264	31200
Core element 454	Constant	3372	*Vmn2r*, LINE	0.123	11	10	11	348	37092
Core element 462	Constant	2700	LINE, SINE,	0.910	6	7	6	40	16200
Core element 484	Constant	3000	LINE, Simple, SINE	0.839	4	4	4	54	12000
AVERAGE (Constant)		3115		0.545	8	8	8	172	23859
Core element 103.1	CNV	1802	LTR, SINE	0.959	14	20	19	127	25228
Core element 103.2	CNV	1686	*Zfp* , LINE, LTR	0.225	8	15	12	129	13488
Core element 146	CNV	2877	LINE, SINE	0.979	19	33	26	103	54663
Core element 154	CNV	2691	LTR, SINE	0.749	24	30	28	236	64584
Core element 177.1	CNV	3900	Simple, *Zfp*	0.110	23	28	27	112	89700
Core element 182	CNV	2629	LINE, LTR, MurSatRep1	0.622	20	27	25	305	52580
Core element 364	CNV	1468	SINE, LTR	0.343	13	16	18	190	19084
Core element 447	CNV	2248	LTR, SINE, Simple/Sat	0.768	24	35	30	108	53952
Core element 510	CNV	2735	LINE, Simple/Sat	0.094	13	19	16	348	35555
Core element 541	CNV	3195	Simple/Sat, *Zfp*	0.076	14	21	20	413	44730
AVERAGE (CNV)		2523		0.492	17	24	22	207	45356

^a^ Detailed information on the known repeats and RefSeq genes that were detected in each core element is provided in Additional file 5. LTR retrotransposons are underlined. Core elements with large CNV were selected by two criteria: 1) a P value lower than the significance value; 2) the 10 core elements that showed the greatest variation among strains. ^b^ Proportion of each core element that comprised the known repeat. The proportion was calculated by dividing the total length of the known repeat in the core element by the total length of the core element. ^c^ Expected copy numbers in B6 were calculated from the number of homologous sequences in each of the 60 groups (Additional file 5). Expected copy numbers in BLG2 and MSM were calculated from the average of the aCGH values and the copy number in B6 (Additional file 7).

## Discussion

It has been reported that the mammalian genome contains many complex arrays of repetitive sequences in the centromeric and subtelomeric regions, as well as other SD regions in which many repetitive sequences coexist in a complex manner [30,31]. However, many complex arrays of repetitive sequences, in particular large SD regions, have been neglected during the detailed characterization of genome structure, partly owing to the lack of an appropriate method for the comprehensive analysis of such highly complex structures. In the present study, we conducted a whole-genome search for complex arrays of repetitive regions by the self-comparative-plot method using the SHEAP program. The advantages of SHEAP are: (i) its applicability to massively long sequences (i.e., whole genome sequences of human or mouse), (ii) its applicability to sequences that contain many global repetitive structures, and (iii) its ability to complete the analysis within a reasonable time frame. With respect to the last point, SHEAP can complete the self-comparison of one human or mouse chromosome within 20 minutes when using a conventional personal computer. As a result, in this study, it was possible to visualize remarkably large SD regions, which covered more than 500 kbp and were composed of complex arrays of duplicated sequences in both forward and reverse directions, as square dark patches.

The mouse genome has been systematically searched for regions that contain SDs [18]. All of the large SD regions that were identified in the present study were also reported as SD regions in an earlier study [18]. However, other SD regions that were reported previously, such as those on chromosomes 1, 6, 8, and 17, were not detected as dark square patches in the self-comparative-plots of whole chromosomes that were generated by SHEAP. The results indicate a limitation of this approach based on self-comparative plots because dark square patches were not apparent in some SD regions. Nevertheless, they were detected at higher magnification (Additional file 9), and showed different patterns to those of the dark square patches. These results suggest that different types of SD exist in the mouse genome. Indeed, in the self-comparative-plot analysis of sequence similarity among large SD regions (Additional file 10), we found that most of the SDs comprised unique repetitive sequences, although all of the SDs share many known repetitive elements. Furthermore, this observation suggests that interchromosomal nonallelic homologous recombination has occurred rarely among the SDs in the mouse genome, consistent with a previously described finding [18], and that the SDs have formed and evolved independently.

The present study is the first detailed analysis of repetitive elements in SD13M, which is one of the large SDs of the mouse genome. The results showed that six core elements within SD13M contained the functionally uncharacterized satellite repeat MurSatRep. The presence of this satellite repeat was characteristic of SD13M because its frequency was greater in SD13M than in SDs overall (Table 1). The transposable element MurSatRep1 is presumed to be associated with pericentromeric duplications (Repbase database). These results support the contention that core elements might have structural significance, similar to repetitive sequences in the centromeric region [32,33]. In addition, four core elements contained MMSAT4, which has been reported to be a satellite sequence that encodes zinc finger proteins. The presence of this repeat was also characteristic of SD13M, but its function is unknown. Other core elements contained known repetitive elements such as LINEs, small regions of RefSeq sequences, and LTR sequences. LINEs are known to be enriched in intermediately sized and larger SDs (>10 kbp) and duplicated gene regions, and are supposed to facilitate nonallelic homologous recombination [7,18,34,35]. The existence of these repetitive elements in the core elements strongly supports the hypothesis that SD13M was formed by combinations of nonallelic homologous recombination events. Furthermore, regions with abundant transposable elements are thought to be targeted preferentially by other transposition events [35]. The presence of a higher proportion of LTR sequences in CNV-type than in constant-type core elements suggests that retrotransposition of LTRs also promotes nonallelic homologous recombination and caused CNV in SD13M. This model is very similar to the case of centromere expansion in rice, in which retrotransposons and satellite repeats were duplicated by intra-element homologous recombination [36].

## Conclusions

In the present study, we characterized both the structures and the relative quantities of the repetitive elements in a complex SD region on chromosome 13 of mouse. Although we did not address the functional significance of SDs in this study, their characteristic repetitive structure indicates that they are similar to the functionally important centromeric region [32,33]. Interestingly, SD13M is included in the region of chromosome 13 that was difficult to substitute from strain B6 to MSM during the course of establishing a consomic strain [21]. The results of structural and quantitative analyses in this study may help to elucidate the biological role of SD13M.

## Methods

### Self-plot analysis

In order to detect SDs, we conducted self-plot analysis using genome sequence data (Jul. 2007 assembly of the mouse genome, mm9, NCBI Build37). The Y chromosome was excluded from the analysis because the sequence data for this chromosome included uncertain nucleotides at the level of 83.0%. SDs were visualized and detected using SHEAP, an algorithm capable of efficient discovery of similar short substrings [19]. SHEAP can draw a self-comparative-plot more rapidly than harplot or BLAST-based programs [37-39]. The regions that contained SDs were detected simply as clusters of dots that formed complex diagonal lines on images of Figure 1. For the rough detection and visualization of SDs in each chromosome, the criteria of the SHEAP analysis were set to assign a dot to a pixel whenever a pair of sequences of 3,000 bp shared more than three 30-bp homologous sequences (≤2 mismatches), and the overall distance between the paired sequences was larger than 300 bp.For the detailed analysis of repetitive elements in SDs, dots were assigned to pairs of 300-bp sequences when they shared at least one 30-bp homologous sequence (≤2 mismatches).

### Database analysis

For further detailed analysis of SD13M, we used sequence data from B6 (NCBI Build37). Before conducting further analysis, we checked assembly data of BAC contig, and found no apparent errors (data not shown). Although we cannot rule out the possibility of small errors, the overall sequence is reliable. The detection of known repeats and masking of SD13M were conducted with Repeatmasker (downloaded from http://www.repeatmasker.org/) using Repeatmaskerlibraries -20090604 (downloaded from http://www.girinst.org/, megablast –p megablast –W 28 –G 0 –E 0 –q -2 –i filenameA –jfilenameB). The pairwise alignments of fundamental repetitive sequences were conducted using bl2seq (bl2seq –ifilenameA –jfilenameB –p blastn –FF). Known repeats were characterized using Repbase (http://www.girinst.org/repbase/). All RefSeq genes in masked core elements were identified with BLASTN (2.2.24+) [40-42] using the RefSeqGene database (*Mus musculus*, NCBI Transcript Reference Sequences). A MUSCLE analysis was conducted through a web site (http://www.ebi.ac.uk/Tools/msa/muscle/) in March 2011.

### Mouse strains

Three inbred strains of mouse, BLG2/Ms (BLG2), C57BL/6J (B6), and MSM/Ms (MSM), were maintained in the animal facility at the National Institute of Genetics (NIG), Mishima, Japan. Both BLG2 and MSM were established as inbred strains after 20 generations of brother–sister mating [43,44]. The BLG2 and MSM strains belong to the musculus subspecies group, whereas B6 belongs to the domesticus subspecies group [45]. All mice were kept in accordance with NIG guidelines, and all procedures were carried out with approval (No. 18–18 and 19–6) from the Committee for Animal Care and Use of the NIG.

### Comparative genome hybridization array (aCGH)

To conduct aCGH analysis on the SD13M region, we designed four types of custom tiling array probe. The first and second types of probe covered a region of approximately 6 Mbp that surrounded SD13M (63,267,529–69,226,366; NCBI Build37). Probes of the first type were completely unique within that genomic region, whereas the second type of probe appeared more than twice in the genomic region covered, but did not appear in other genomic regions. When the first and second types of probe were combined, the average interval between them was 46.3 bp. These probes should detect CNV only in SD13M. The third type of probe covered a small area of chromosome 17 (80,000,245–80,099,784, NCBI Build37) that does not contain an SD and was used for normalization. Owing to the fact that the probes were isothermal, the lengths of the probes ranged from 50 to 75 bp. All of the probes were arrayed in triplicate. The total number of probes in an array was 75,000 (25,000 types of probe × 3). As a result of the aCGH analysis, a total of 9,929 types of probe were mapped on 53 core elements. It was not possible to map a sufficient number of probes on the remaining seven core elements because appropriate sequence probes for the aCGH tiling array were not well represented on these elements (number of probes/core element: < 30).

Genomic DNA was purified from the nuclei of kidney cells from B6, MSM, and BLG mice, and then purified further with DNeasy (Qiagen). Reference DNA (B6) and test DNA (BLG2 or MSM) samples were labeled differentially with Cy3 and Cy5, respectively, and hybridized competitively to a microarray chip. Labeling and hybridization were carried out by a commercial aCGH service (Nimblegen Systems, Roche). The fluorescence ratio between Cy3 and Cy5 was normalized against the average value for the control probes designed for chromosome 17.Four sets of aCGH analysis were conducted between two strain pairs, BLG2 and B6, and B6 and MSM. Each genomic DNA sample had two biological replicates. Relative CNV values as compared with B6 are described as the log2 values for each probe on SD13M. Given that most of the probes showed a higher copy number in the BLG2 and MSM strains than in B6, the strain that was the source of the sequence information, the difference of the CNV values was unlikely to have been caused by sequence polymorphisms.

### Quantitative PCR (qPCR) analysis using genomic DNA

Primers for qPCR of the core elements were designed by a web-based service, PRIMER3 (http://frodo.wi.mit.edu/primer3/), using a mispriming library (RODENT_AND_SIMPLE). A single-copy-number gene, parathyroid hormone-related protein (Pthlh, NM_08970), was used to normalize the levels of genomic DNA [46]. The sequences of all the primers used in the present study are listed in Additional file 7. The qPCR on the genomic DNA of B6, BLG, and MSM mice was conducted using SYBR^®^ Premix Ex Taq™ II (TAKARA) and a Thermal Cycler Dice Real Time System (TAKARA), in accordance with the manufacturer’s instructions. All reactions were carried out with biological triplicates, each with experimental duplicates. Relative comparative threshold cycle (Ct) values were calculated on the basis of the second derivative maximum method using dedicated software (TAKARA TP800). Relative copy numbers of core elements were estimated by comparison with other strains or genotypes on the basis of the Ct values. Genomic DNA was prepared from different versions of consomic strain B6-Chr13A^MSM^, which contains entire chromosome 13 of MSM in a B6 genetic background. The homozygotes of entire chromosome 13 infrequently appeared in the crosses of the heterozygotes for chromosome 13 of MSM and B6. The different versions were homozygous or heterozygous for the MSM allele of the SD13M region (SD13M^MSM/MSM^ and SD13M^MSM/B6^, respectively), or homozygous for the B6 allele (SD13M^B6/B6^). By using genomic DNA from these strains, we were able to investigate the relative copy number of core elements by targeting the SD13M region. The genotypes of these consomic mice are shown in Additional file 7.

### Divergence within each group of core elements

The pairwise divergences of the homologous sequences in each group of core elements were calculated by Repeatmasker using custom-made Repeatmasker library files that contained each of the homologous sequences. The divergence of a core element group was represented by the average of these pairwise divergences. Average divergences were calculated for the seven core elements with constant copy number and for the 46 core elements with CNVs. Core elements 177 and 254 were excluded from the analysis because their sequences were partially contained within core elements 541 and 244, respectively.

### Programs and statistics

SHEAP is available online (http://research.nii.ac.jp/~uno/codes.htm). All programs, including SHEAP, SHEPHERD, and a pair-comparison program based on BLAST, are available upon request. Free software, R (http://www.r-project.org/), was used for graphics and statistical analysis.

For the analysis of CNV in core elements, the significance of differences in copy number was determined by a simple two-sided t-test. When the average of the aCGH values (log2) mapped on each core element was zero, the null hypothesis of no difference in copy number between two strains was applied. Thus, t-statistics were calculated using the formula $\sqrt{n\left(i\right)/U\left(i\right)}$$\bar{X}$ (i), where *n(i)* indicates the number of probes, and *U(i)* and $\bar{X}$*(i)* indicate unbiased estimates of the population variance and the average of the aCGH values mapped on the core elements, respectively. The P value for each core element was calculated by t-statistics with a t-distribution [df = (probe number) –1] under the null hypothesis. We adjusted the P value for multiple comparisons (Bonferroni, N = 54), and when it was below the threshold for significance (<0.05), the core element was interpreted as having significant CNV.

## Abbreviations

CNV: Copy number variation; CNVR: CNV region; SD: Segmental duplication; LINE: Long interspersed nuclear element; aCGH: Comparative genome hybridization array; LTR: Long terminal repeat; bp: Base pairs; Chr: Chromosome; MSM: MSM/Ms; B6: C57BL/6J; SINE: Short interspersed nuclear element; Zfp: Zinc finger protein; Vmn2r: Vomeronasal 2 receptor; Cbx3: Chromobox homolog 3.

## Competing interests

The authors declare that they have no competing interests.

## Authors’ contributions

JU collected and analyzed the data generated from the aCGH and qPCR experiments. AM developed SHEPHERD and conducted computational analyses, which included core element extraction and the application of aCGH values to core elements. KI analyzed known repeats. TU developed SHEAP and conducted the self-plot analysis. TK supervised the project. JU wrote the initial draft and all authors edited the manuscript and approved its final version. All authors read and approved the final manuscript.

## Supplementary Material

Additional file 1 **Self-plot of all mouse chromosomes. **Detection of large SDs in self-plots of chromosomes 1 to 19 and chromosome X. Click here for file

Additional file 2 **Output of self-comparative plot analysis for chromosome 13. **Dot-plot matrix showing candidate SD as a very small square dot or even a small intense dot. However, at a larger magnification, square dark patches became more obvious, and many diagonal split lines became visible. Click here for file

Additional file 3 **Sequence information of identified core elements.** The group, position, proportion of matched sequence, and sequences of homologous regions were determined for core elements in the SD13M region. Click here for file

Additional file 4 **Annotation of the core elements. **Known repeats of RefSeq genes were detected using RepeatMasker and RefSeq BLAST. Click here for file

Additional file 5 **Mapped aCGH values and divergence of core elements. **Lengths of core elements, information of known repeats in the core element, and number of probes and P values obtained by analysis of aCGH values using the t-test. Click here for file

Additional file 6 **Size and number of RefSeq genes within the core elements. **The information on core elements that contained partial RefSeq genes, including *Cbx3*, *Vmn2r*, and *Zfp*. Click here for file

Additional file 7 **Materials for quantitative PCR. **The genotypes of B6-Chr13A^MSM^ consomic mice and the primer sequences used for quantitative PCR are shown. Click here for file

Additional file 8 **Results of quantitative PCR analysis. **Relative copy number values were determined by qPCR analysis of genomic DNA from consomic B6-Chr13A^MSM^. Click here for file

Additional file 9 **Detection of large SD regions at higher magnification of the self-plot. **SD regions on chromosomes 1, 6, 8, and 17, detected as dark square patches at higher magnification of the self-plot. Click here for file

Additional file 10 **Analysis of sequence similarity among large SD regions. **No similarity was observed in large SDs except for a large number of known repetitive sequences. Click here for file
