# Supplementary material for: Identification of both copy number variation-type and constant-type core elements in a large segmental duplication region of the mouse genome
Source: BMC Genomics. 2013 Jul 8;14:455. doi: 10.1186/1471-2164-14-455 (PMC3722088; doi:10.1186/1471-2164-14-455)
Supplement: Additional file 1 — Self-plot of all mouse chromosomes. Detection of large SDs in self-plots of chromosomes 1 to 19 and chromosome X. [file 1471-2164-14-455-S1.pdf]

Additional file 1. Self-comparative-plots of all mouse chromosomes.

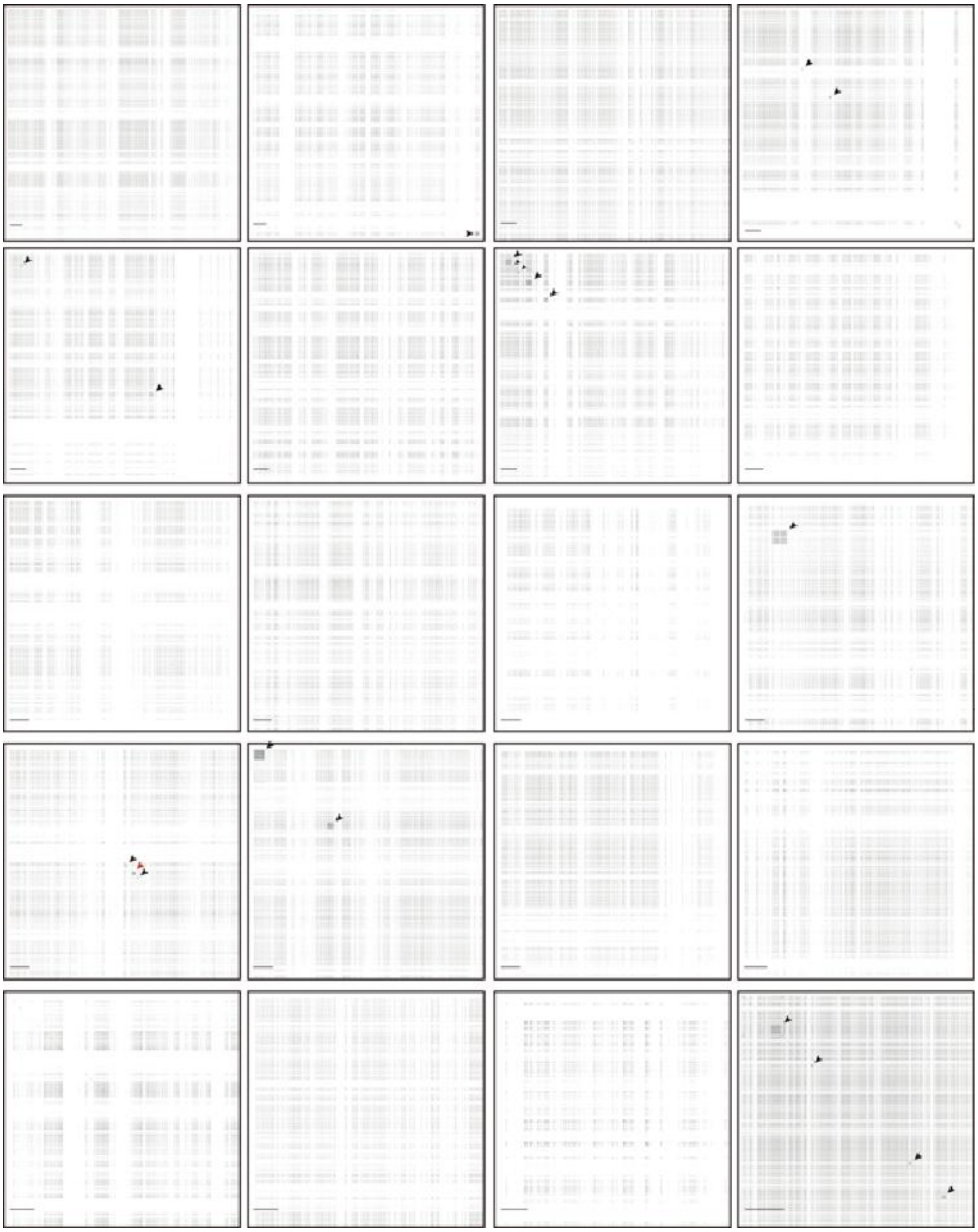

Self-comparative-plots of chromosomes 1 to 19 and Chr X are shown in order from the upper left to lower right. The result for Chr Y was excluded because a large proportion of the sequence of this chromosome has not been determined. The size of each chromosome (Mbp) is given below: Chr. 1, 197; Chr. 2, 182; Chr. 3, 160; Chr. 4, 156; Chr. 5, 153; Chr. 6, 150; Chr. 7, 153; Chr. 8, 132; Chr. 9, 124; Chr. 10, 130; Chr. 11, 122; Chr. 12, 121; Chr. 13, 120; Chr. 14, 125; Chr. 15, 103; Chr. 16, 98; Chr. 17, 95; Chr. 18, 91; Chr. 19, 61; Chr. X, Undetermined (NCBI Build 37.1). Arrowheads indicate large size SDs (>500 kbp) shown in Figure 1. The red arrowhead indicates a complex CNVR associated with an SD in the middle region of Chr. 13 (SD13M). The diagonal lines from top left to bottom right, which indicate a complete match between two paired chromosomal sequences, were eliminated by the algorithm in each self-comparative-plot. Bars indicate 10 Mbp.
