# Supplementary material for: Identification of both copy number variation-type and constant-type core elements in a large segmental duplication region of the mouse genome
Source: BMC Genomics. 2013 Jul 8;14:455. doi: 10.1186/1471-2164-14-455 (PMC3722088; doi:10.1186/1471-2164-14-455)
Supplement: Additional file 6 — Size and number of RefSeq genes within the core elements. The information on core elements that contained partial RefSeq genes, including Cbx3, Vmn2r, and Zfp. [file 1471-2164-14-455-S6.pdf]

Additional file 6. Size and number of RefSeq genes within the core elements.

	Size				Number of CoreElements in SD13M			
	Average (bp)	Average Ratio in CoreElement	Min (bp)	Max (bp)	B6	BLG2	MSM	Total length (bp)
Cbx3	1119	0.37	-	-	5	5	5	5595
Vmn2r	486	0.16	93	997	29	30	31	14101
Zfp	108	0.08	31	148	108	146	134	14122
Others	227	0.03	83	370	5	7	7	1135
misc or ncRNA	469	0.15	62	917	40	42	43	18768
							Whole RefSeq	53721

The number of core elements that contained partial RefSeq genes, including *Cbx3*, *Vmn2r*, *Zfp* and other genes, is listed. The average, minimum and maximum size, and ratio of RefSeq in each core element are also listed. The copy numbers of core elements in BLG2 and MSM were calculated on the basis of the copy numbers reported in the B6 genome database and the mapped aCGH values (Additional file 5). Core elements 177 and 254 were excluded from these analyses because their sequences were contained within core element 541 and 244, respectively.
