# Supplementary material for: Identification of both copy number variation-type and constant-type core elements in a large segmental duplication region of the mouse genome
Source: BMC Genomics. 2013 Jul 8;14:455. doi: 10.1186/1471-2164-14-455 (PMC3722088; doi:10.1186/1471-2164-14-455)
Supplement: Additional file 7 — Materials for quantitative PCR. The genotypes of B6-Chr13AMSM consomic mice and the primer sequences used for quantitative PCR are shown. [file 1471-2164-14-455-S7.pdf]

Additional file 7. Materials for quantitative PCR

Genotypes of B6-Chr13A^{MSM} consomic mice.

	D13Mit21	D13Mit186	D13Mit283	D13Mit311	D13Mit310	D13Mit66	D13Mit281	D13Mit9
Position	55673902-55674065	59775006-59775152	63398675-63398787	63750301-63750421	65062945-65063066	67271283-67271431	68854945-68855043	81241701-81241825
N7F14	M / M	M / M	B / B	B / B	B / B	B / B	M / M	M / M
N7F14	M / M	M / M	B / B	B / B	B / B	B / B	M / M	M / M
N7F14	M / M	M / M	B / B	B / B	B / B	B / B	M / M	M / M
N7F14	M / M	M / M	B / M	B / M	B / M	B / M	M / M	M / M
N7F14	M / M	M / M	B / M	B / M	B / M	B / M	M / M	M / M
N7F14	M / M	M / M	B / M	B / M	B / M	B / M	M / M	M / M
N7F14	M / M	M / M	M / M	M / M	M / M	M / M	M / M	M / M
N7F13	M / M	M / M	M / M	M / M	M / M	M / M	M / M	M / M
N7F14	M / M	M / M	M / M	M / M	M / M	M / M	M / M	M / M

B6-Chr13A^{MSM} consomic mice, which have homozygous B6 alleles and heterozygous and homozygous MSM alleles in the middle region of Chr 13, which includes SD13M.

Primer sequences used in quantitative PCR.

Name	Primer_sequence	Start position in CoreElement	Length
CoreElement454_1F	CAGGGAGGAAAGTGATCCAG	464	20
CoreElement454_2R	GGAGCTTCACTGTGGCTTTC	577	20
CoreElement454_3F	TGGGACATTAACAGATATGGCTA	2570	23
CoreElement454_4R	TGCACTGCAGGGTAATACAAA	2662	18
CoreElement541_1F	GCCAGCCATGTCCCTTATT	320	19
CoreElement541_2R	ATCAGCAAAAGGCTGGTGTT	456	20
CoreElement541_3F	TGGTGAAGACACATGGCCTA	1599	20
CoreElement541_4R	TGGGGAATCCTCAAAAGATG	1693	20
CoreElement541_5F	TTACCCCCAACCTCTTACCC	2050	20
CoreElement541_6R	TAGGCGATTGGAGGGTTATG	2162	20
Pthlh_qPCR_F	GAACACCCGCGTTTGAAGAG		
Pthlh_qPCR_R	GCTGTGGCTCCCATAGCAA		

Start position indicates the position of the primers with respect to the core elements.
