# Supplementary material for: Identification of both copy number variation-type and constant-type core elements in a large segmental duplication region of the mouse genome
Source: BMC Genomics. 2013 Jul 8;14:455. doi: 10.1186/1471-2164-14-455 (PMC3722088; doi:10.1186/1471-2164-14-455)
Supplement: Additional file 8 — Results of quantitative PCR analysis. Relative copy number values were determined by qPCR analysis of genomic DNA from consomic B6-Chr13AMSM. [file 1471-2164-14-455-S8.pdf]

Additional file 8. Quantitative PCR analysis using genomic DNA from consomic B6-Chr13A^{MSM}.

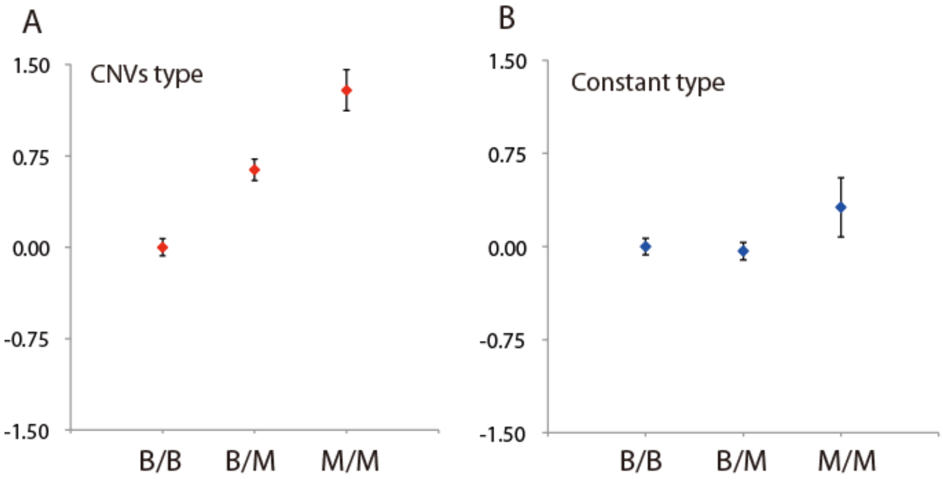

(A) Relative copy number values quantified by qPCR increased as the dosage of the MSM allele in B6-Chr13A^{MSM} increased. This result showed that the copy number of the MSM allele for CoreElement541 was higher than that of the B6 allele. Primers designed against nucleotides 1771–1844 of core element 541 were used for qPCR analysis.

(B) The relative copy number quantified by qPCR was constant when the dosage of the MSM allele for core element 454 was increased in B6-Chr13A^{MSM}. This result shows that the copy numbers of the B6 and MSM alleles for core element 454 are nearly equal. Primers designed against nucleotides 1771–1844 of core element 454 were used for the qPCR analysis. (*** $P < 0.001$)
