# Supplementary material for: Identification of both copy number variation-type and constant-type core elements in a large segmental duplication region of the mouse genome
Source: BMC Genomics. 2013 Jul 8;14:455. doi: 10.1186/1471-2164-14-455 (PMC3722088; doi:10.1186/1471-2164-14-455)
Supplement: Additional file 9 — Detection of large SD regions at higher magnification of the self-plot. SD regions on chromosomes 1, 6, 8, and 17, detected as dark square patches at higher magnification of the self-plot. [file 1471-2164-14-455-S9.pdf]

Additional file 9. Detection of large SD regions in higher magnification of the self-comparative-plots

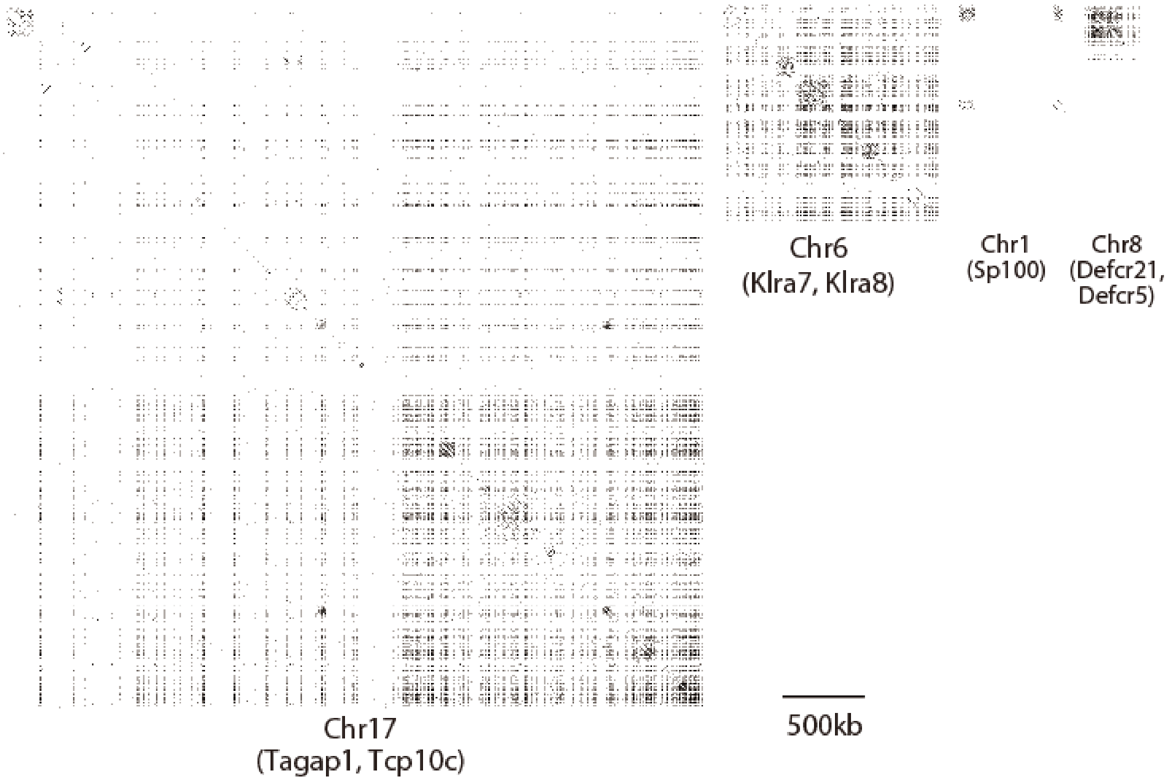

SD regions on Chrs 1, 6, 8, and 17, which have been reported previously (She et al. 2008), were not detected as dark square patches in self-comparative-plots of whole chromosomes in this study. However, these SD regions were detected in further magnification, indicating
