# Supplementary material for: Identification of both copy number variation-type and constant-type core elements in a large segmental duplication region of the mouse genome
Source: BMC Genomics. 2013 Jul 8;14:455. doi: 10.1186/1471-2164-14-455 (PMC3722088; doi:10.1186/1471-2164-14-455)
Supplement: Additional file 10 — Analysis of sequence similarity among large SD regions. No similarity was observed in large SDs except for a large number of known repetitive sequences. [file 1471-2164-14-455-S10.pdf]

Additional file 10. Sequence similarity among large SD regions

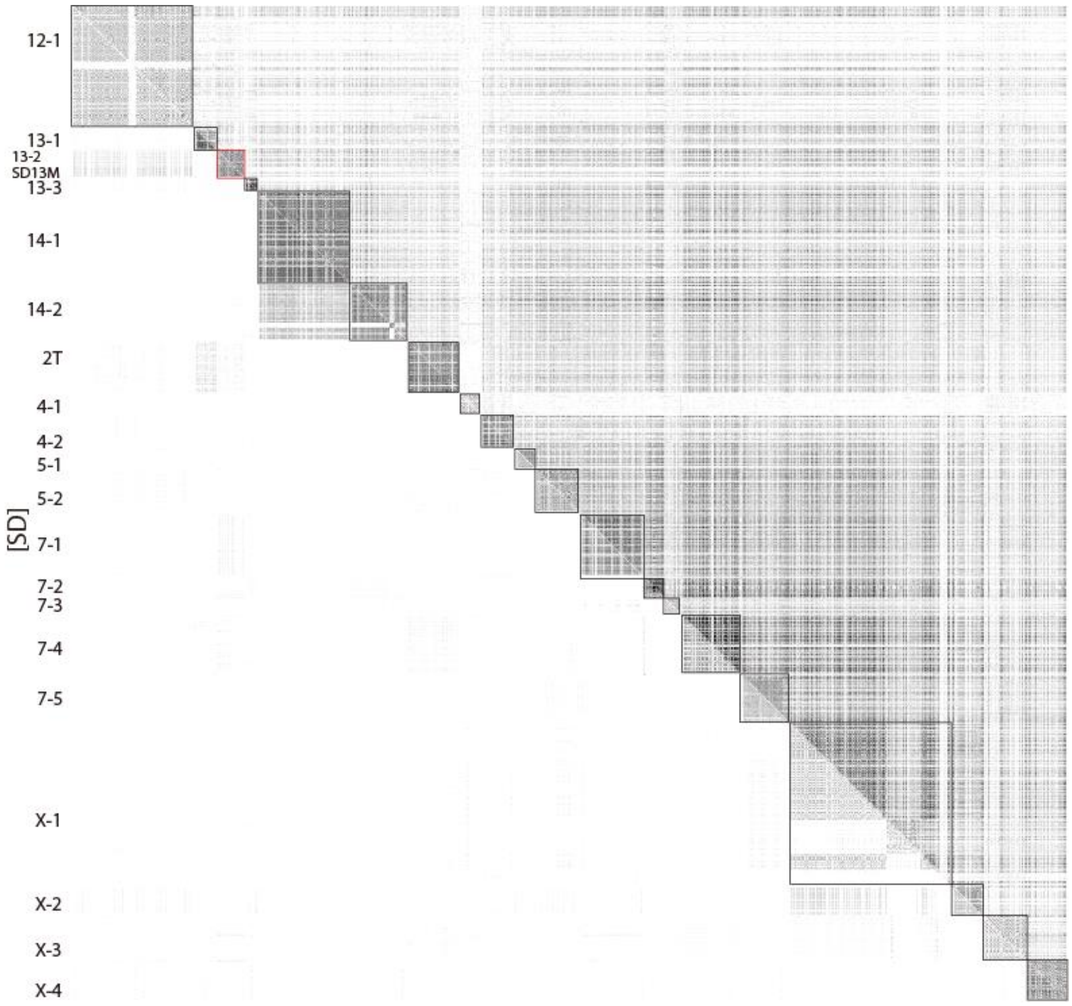

Sequence similarity among large SD regions (>500 kbp) shown in Figure 1 was investigated with SHEAP to draw self-comparative-plot. Entire sequences of all the large SD regions were connected to produce single large sequence. Then, the connected sequence was applied to the self-comparative-plot analysis. The lower left triangle shows a self-comparative-plot of the sequence after known repeat sequences have been masked by using RepeatMasker while the upper right triangle shows a self-comparative-plot of the intact sequence. The black boxes indicate the regions of large SDs. The red box indicates SD13M. Many dots, which represent homologous sequences, are shared among the intact SD sequences but homology was not apparent when the known repetitive sequences were eliminated from the sequence data. The results indicated that most of large SDs have no similarity except for a large number of known repetitive sequences.
